# The future of digital health with federated learning

**DOI:** 10.1038/s41746-020-00323-1

**Published:** 2020-09-14

**Authors:** Nicola Rieke, Jonny Hancox, Wenqi Li, Fausto Milletarì, Holger R. Roth, Shadi Albarqouni, Spyridon Bakas, Mathieu N. Galtier, Bennett A. Landman, Klaus Maier-Hein, Sébastien Ourselin, Micah Sheller, Ronald M. Summers, Andrew Trask, Daguang Xu, Maximilian Baust, M. Jorge Cardoso

**Affiliations:** 1 NVIDIA GmbH, Munich, Germany; 2 Technical University of Munich (TUM), Munich, Germany; 3 NVIDIA Ltd, Reading, UK; 4 NVIDIA Ltd, Cambridge, UK; 5 NVIDIA Corporation, Bethesda, USA; 6 Imperial College London, London, UK; 7 University of Pennsylvania (UPenn), Philadelphia, PA USA; 8 Owkin, Paris, France; 9 Vanderbilt University, Nashville, TN USA; 10 German Cancer Research Center (DKFZ), Heidelberg, Germany; 11 Heidelberg University Hospital, Heidelberg, Germany; 12 King’s College London (KCL), London, UK; 13 Intel Corporation, Santa Clara, CA USA; 14 Clinical Center, National Institutes of Health (NIH), Bethesda, MD USA; 15 OpenMined, Oxford, UK; 16 University of Oxford, Oxford, UK; 17 Centre for the Governance of AI (GovAI), Oxford, UK

**Keywords:** Medical research, Medical imaging

## Abstract

Data-driven machine learning (ML) has emerged as a promising approach for building accurate and robust statistical models from medical data, which is collected in huge volumes by modern healthcare systems. Existing medical data is not fully exploited by ML primarily because it sits in data silos and privacy concerns restrict access to this data. However, without access to sufficient data, ML will be prevented from reaching its full potential and, ultimately, from making the transition from research to clinical practice. This paper considers key factors contributing to this issue, explores how federated learning (FL) may provide a solution for the future of digital health and highlights the challenges and considerations that need to be addressed.

## Introduction

Research on artificial intelligence (AI), and particularly the advances in machine learning (ML) and deep learning (DL)^1^ have led to disruptive innovations in radiology, pathology, genomics and other fields. Modern DL models feature millions of parameters that need to be learned from sufficiently large curated data sets in order to achieve clinical-grade accuracy, while being safe, fair, equitable and generalising well to unseen data^2–5^.

For example, training an AI-based tumour detector requires a large database encompassing the full spectrum of possible anatomies, pathologies, and input data types. Data like this is hard to obtain, because health data is highly sensitive and its usage is tightly regulated^6^. Even if data anonymisation could bypass these limitations, it is now well understood that removing metadata such as patient name or date of birth is often not enough to preserve privacy^7^. It is, for example, possible to reconstruct a patient’s face from computed tomography (CT) or magnetic resonance imaging (MRI) data^8^. Another reason why data sharing is not systematic in healthcare is that collecting, curating, and maintaining a high-quality data set takes considerable time, effort, and expense. Consequently such data sets may have significant business value, making it less likely that they will be freely shared. Instead, data collectors often retain fine-grained control over the data that they have gathered.

**Fig. 1:**
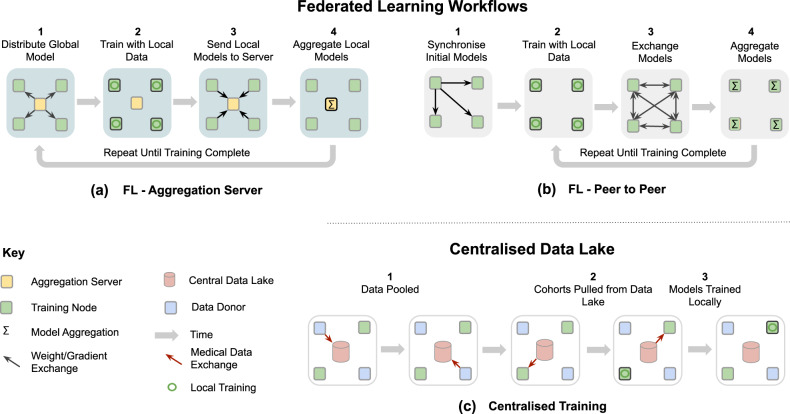
Example federated learning (FL) workflows and difference to learning on a Centralised Data Lake. **a** FL aggregation server—the typical FL workflow in which a federation of training nodes receive the global model, resubmit their partially trained models to a central server intermittently for aggregation and then continue training on the consensus model that the server returns. **b** FL peer to peer—alternative formulation of FL in which each training node exchanges its partially trained models with some or all of its peers and each does its own aggregation. **c** Centralised training—the general non-FL training workflow in which data acquiring sites donate their data to a central Data Lake from which they and others are able to extract data for local, independent training.

Federated learning (FL)^9–11^ is a learning paradigm seeking to address the problem of data governance and privacy by training algorithms collaboratively without exchanging the data itself. Originally developed for different domains, such as mobile and edge device use cases^12^, it recently gained traction for healthcare applications^13–20^. FL enables gaining insights collaboratively, e.g., in the form of a consensus model, without moving patient data beyond the firewalls of the institutions in which they reside. Instead, the ML process occurs locally at each participating institution and only model characteristics (e.g., parameters, gradients) are transferred as depicted in Fig. 1. Recent research has shown that models trained by FL can achieve performance levels comparable to ones trained on centrally hosted data sets and superior to models that only see isolated single-institutional data^16,17^.

A successful implementation of FL could thus hold a significant potential for enabling precision medicine at large-scale, leading to models that yield unbiased decisions, optimally reflect an individual’s physiology, and are sensitive to rare diseases while respecting governance and privacy concerns. However, FL still requires rigorous technical consideration to ensure that the algorithm is proceeding optimally without compromising safety or patient privacy. Nevertheless, it has the potential to overcome the limitations of approaches that require a single pool of centralised data.

We envision a federated future for digital health and with this perspective paper, we share our consensus view with the aim of providing context and detail for the community regarding the benefits and impact of FL for medical applications (section “Data-driven medicine requires federated efforts”), as well as highlighting key considerations and challenges of implementing FL for digital health (section “Technical considerations”).

## Data-driven medicine requires federated efforts

ML and especially DL is becoming the de facto knowledge discovery approach in many industries, but successfully implementing data-driven applications requires large and diverse data sets. However, medical data sets are difficult to obtain (subsection “The reliance on data”). FL addresses this issue by enabling collaborative learning without centralising data (subsection “The promise of federated efforts”) and has already found its way to digital health applications (subsection “Current FL efforts for digital health”). This new learning paradigm requires consideration from, but also offers benefits to, various healthcare stakeholders (section “Impact on stakeholders”).

### The reliance on data

Data-driven approaches rely on data that truly represent the underlying data distribution of the problem. While this is a well-known requirement, state-of-the-art algorithms are usually evaluated on carefully curated data sets, often originating from only a few sources. This can introduce biases where demographics (e.g., gender, age) or technical imbalances (e.g., acquisition protocol, equipment manufacturer) skew predictions and adversely affect the accuracy for certain groups or sites. However, to capture subtle relationships between disease patterns, socio-economic and genetic factors, as well as complex and rare cases, it is crucial to expose a model to diverse cases.

The need for large databases for AI training has spawned many initiatives seeking to pool data from multiple institutions. This data is often amassed into so-called Data Lakes. These have been built with the aim of leveraging either the commercial value of data, e.g., IBM’s Merge Healthcare acquisition^21^, or as a resource for economic growth and scientific progress, e.g., NHS Scotland’s National Safe Haven^22^, French Health Data Hub^23^, and Health Data Research UK^24^.

Substantial, albeit smaller, initiatives include the Human Connectome^25^, the UK Biobank^26^, the Cancer Imaging Archive (TCIA)^27^, NIH CXR8^28^, NIH DeepLesion^29^, the Cancer Genome Atlas (TCGA)^30^, the Alzheimer’s Disease Neuroimaging Initiative (ADNI)^31^, as well as medical grand challenges^32^ such as the CAMELYON challenge^33^, the International multimodal Brain Tumor Segmentation (BraTS) challenge^34–36^ or the Medical Segmentation Decathlon^37^. Public medical data is usually task- or disease-specific and often released with varying degrees of license restrictions, sometimes limiting its exploitation.

Centralising or releasing data, however, poses not only regulatory, ethical and legal challenges, related to privacy and data protection, but also technical ones. Anonymising, controlling access and safely transferring healthcare data is a non-trivial, and sometimes impossible task. Anonymised data from the electronic health record can appear innocuous and GDPR/PHI compliant, but just a few data elements may allow for patient reidentification^7^. The same applies to genomic data and medical images making them as unique as a fingerprint^38^. Therefore, unless the anonymisation process destroys the fidelity of the data, likely rendering it useless, patient reidentification or information leakage cannot be ruled out. Gated access for approved users is often proposed as a putative solution to this issue. However, besides limiting data availability, this is only practical for cases in which the consent granted by the data owners is unconditional, since recalling data from those who may have had access to the data is practically unenforceable.

### The promise of federated efforts

The promise of FL is simple—to address privacy and data governance challenges by enabling ML from non-co-located data. In a FL setting, each data controller not only defines its own governance processes and associated privacy policies, but also controls data access and has the ability to revoke it. This includes both the training, as well as the validation phase. In this way, FL could create new opportunities, e.g., by allowing large-scale, in-institutional validation, or by enabling novel research on rare diseases, where the incident rates are low and data sets at each single institution are too small. Moving the model to the data and not vice versa has another major advantage: high-dimensional, storage-intense medical data does not have to be duplicated from local institutions in a centralised pool and duplicated again by every user that uses this data for local model training. As the model is transferred to the local institutions, it can scale naturally with a potentially growing global data set without disproportionately increasing data storage requirements.

**Fig. 2:**
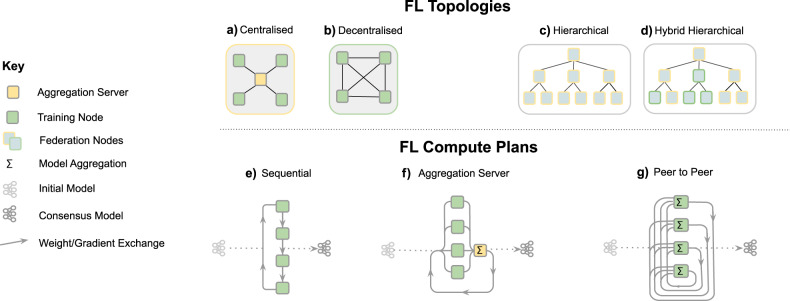
Overview of different FL design choices. FL topologies—communication architecture of a federation. **a** Centralised: the aggregation server coordinates the training iterations and collects, aggregates and distributes the models to and from the Training Nodes (Hub & Spoke). **b** Decentralised: each training node is connected to one or more peers and aggregation occurs on each node in parallel. **c** Hierarchical: federated networks can be composed from several sub-federations, which can be built from a mix of Peer to Peer and Aggregation Server federations (**d**)). FL compute plans—trajectory of a model across several partners. **e** Sequential training/cyclic transfer learning. **f** Aggregation server, **g** Peer to Peer.

As depicted in Fig. 2, a FL workflow can be realised with different topologies and compute plans. The two most common ones for healthcare applications are via an aggregation server^16–18^ and peer to peer approaches^15,39^. In all cases, FL implicitly offers a certain degree of privacy, as FL participants never directly access data from other institutions and only receive model parameters that are aggregated over several participants. In a FL workflow with aggregation server, the participating institutions can even remain unknown to each other. However, it has been shown that the models themselves can, under certain conditions, memorise information^40–43^. Therefore, mechanisms such as differential privacy^44,45^ or learning from encrypted data have been proposed to further enhance privacy in a FL setting (c.f. section “Technical considerations”). Overall, the potential of FL for healthcare applications has sparked interest in the community^46^ and FL techniques are a growing area of research^12,20^.

### Current FL efforts for digital health

Since FL is a general learning paradigm that removes the data pooling requirement for AI model development, the application range of FL spans the whole of AI for healthcare. By providing an opportunity to capture larger data variability and to analyse patients across different demographics, FL may enable disruptive innovations for the future but is also being employed right now.

In the context of electronic health records (EHR), for example, FL helps to represent and to find clinically similar patients^13,47^, as well as predicting hospitalisations due to cardiac events^14^, mortality and ICU stay time^19^. The applicability and advantages of FL have also been demonstrated in the field of medical imaging, for whole-brain segmentation in MRI^15^, as well as brain tumour segmentation^16,17^. Recently, the technique has been employed for fMRI classification to find reliable disease-related biomarkers^18^ and suggested as a promising approach in the context of COVID-19^48^.

It is worth noting that FL efforts require agreements to define the scope, aim and technologies used which, since it is still novel, can be difficult to pin down. In this context, today’s large-scale initiatives really are the pioneers of tomorrow’s standards for safe, fair and innovative collaboration in healthcare applications.

These include consortia that aim to advance *academic* research, such as the Trustworthy Federated Data Analytics (TFDA) project^49^ and the German Cancer Consortium’s Joint Imaging Platform^50^, which enable decentralised research across German medical imaging research institutions. Another example is an international research collaboration that uses FL for the development of AI models for the assessment of mammograms^51^. The study showed that the FL-generated models outperformed those trained on a single institute’s data and were more generalisable, so that they still performed well on other institutes’ data. However, FL is not limited just to academic environments.

By linking healthcare institutions, not restricted to research centres, FL can have direct *clinical* impact. The on-going HealthChain project^52^, for example, aims to develop and deploy a FL framework across four hospitals in France. This solution generates common models that can predict treatment response for breast cancer and melanoma patients. It helps oncologists to determine the most effective treatment for each patient from their histology slides or dermoscopy images. Another large-scale effort is the Federated Tumour Segmentation (FeTS) initiative^53^, which is an international federation of 30 committed healthcare institutions using an open-source FL framework with a graphical user interface. The aim is to improve tumour boundary detection, including brain glioma, breast tumours, liver tumours and bone lesions from multiple myeloma patients.

Another area of impact is within *industrial* research and translation. FL enables collaborative research for, even competing, companies. In this context, one of the largest initiatives is the Melloddy project^54^. It is a project aiming to deploy multi-task FL across the data sets of 10 pharmaceutical companies. By training a common predictive model, which infers how chemical compounds bind to proteins, partners intend to optimise the drug discovery process without revealing their highly valuable in-house data.

### Impact on stakeholders

FL comprises a paradigm shift from centralised data lakes and it is important to understand its impact on the various stakeholders in a FL ecosystem.

#### Clinicians

Clinicians are usually exposed to a sub-group of the population based on their location and demographic environment, which may cause biased assumptions about the probability of certain diseases or their interconnection. By using ML-based systems, e.g., as a second reader, they can augment their own expertise with expert knowledge from other institutions, ensuring a consistency of diagnosis not attainable today. While this applies to ML-based system in general, systems trained in a federated fashion are potentially able to yield even less biased decisions and higher sensitivity to rare cases as they were likely exposed to a more complete data distribution. However, this demands some up-front effort such as compliance with agreements, e.g., regarding the data structure, annotation and report protocol, which is necessary to ensure that the information is presented to collaborators in a commonly understood format.

#### Patients

Patients are usually treated locally. Establishing FL on a global scale could ensure high quality of clinical decisions regardless of the treatment location. In particular, patients requiring medical attention in remote areas could benefit from the same high-quality ML-aided diagnoses that are available in hospitals with a large number of cases. The same holds true for rare, or geographically uncommon, diseases, that are likely to have milder consequences if faster and more accurate diagnoses can be made. FL may also lower the hurdle for becoming a data donor, since patients can be reassured that the data remains with their own institution and data access can be revoked.

#### Hospitals and practices

Hospitals and practices can remain in full control and possession of their patient data with complete traceability of data access, limiting the risk of misuse by third parties. However, this will require investment in on-premise computing infrastructure or private-cloud service provision and adherence to standardised and synoptic data formats so that ML models can be trained and evaluated seamlessly. The amount of necessary compute capability depends of course on whether a site is only participating in evaluation and testing efforts or also in training efforts. Even relatively small institutions can participate and they will still benefit from collective models generated.

#### Researchers and AI developers

Researchers and AI developers stand to benefit from access to a potentially vast collection of real-world data, which will particularly impact smaller research labs and start-ups. Thus, resources can be directed towards solving clinical needs and associated technical problems rather than relying on the limited supply of open data sets. At the same time, it will be necessary to conduct research on algorithmic strategies for federated training, e.g., how to combine models or updates efficiently, how to be robust to distribution shifts^11,12,20^. FL-based development implies also that the researcher or AI developer cannot investigate or visualise all of the data on which the model is trained, e.g., it is not possible to look at an individual failure case to understand why the current model performs poorly on it.

#### Healthcare providers

Healthcare providers in many countries are affected by the on-going paradigm shift from volume-based, i.e., fee-for-service-based, to value-based healthcare, which is in turn strongly connected to the successful establishment of precision medicine. This is not about promoting more expensive individualised therapies but instead about achieving better outcomes sooner through more focused treatment, thereby reducing the cost. FL has the potential to increase the accuracy and robustness of healthcare AI, while reducing costs and improving patient outcomes, and may therefore be vital to precision medicine.

#### Manufacturers

Manufacturers of healthcare software and hardware could benefit from FL as well, since combining the learning from many devices and applications, without revealing patient-specific information, can facilitate the continuous validation or improvement of their ML-based systems. However, realising such a capability may require significant upgrades to local compute, data storage, networking capabilities and associated software.

## Technical considerations

FL is perhaps best-known from the work of Konečnỳ et al.^55^, but various other definitions have been proposed in the literature^9,11,12,20^. A FL workflow (Fig. 1) can be realised via different topologies and compute plans (Fig. 2), but the goal remains the same, i.e., to combine knowledge learned from non-co-located data. In this section, we will discuss in more detail what FL is, as well as highlighting the key challenges and technical considerations that arise when applying FL in digital health.

### Federated learning definition

FL is a learning paradigm in which multiple parties train collaboratively without the need to exchange or centralise data sets. A general formulation of FL reads as follows: Let ${\mathcal{L}}$ denote a global loss function obtained via a weighted combination of *K* local losses ${\{{{\mathcal{L}}}_{k}\}}_{k = 1}^{K}$, computed from private data *X*_*k*_, which is residing at the individual involved parties and never shared among them:

1 $$\mathop{\min }\limits_{\phi }{\mathcal{L}}(X;\phi)\quad \,\text{with}\,\quad {\mathcal{L}}(X;\phi)=\mathop{\sum }\limits_{k = 1}^{K}{w}_{k}\ {{\mathcal{L}}}_{k}({X}_{k};\phi),$$

where *w*_*k*_ > 0 denote the respective weight coefficients.

In practice, each participant typically obtains and refines a global consensus model by conducting a few rounds of optimisation locally and before sharing updates, either directly or via a parameter server. The more rounds of local training are performed, the less it is guaranteed that the overall procedure is minimising (Eq. 1)^9,12^. The actual process for aggregating parameters depends on the network topology, as nodes might be segregated into sub-networks due to geographical or legal constraints (see Fig. 2). Aggregation strategies can rely on a single aggregating node (hub and spokes models), or on multiple nodes without any centralisation. An example is peer-to-peer FL, where connections exist between all or a subset of the participants and model updates are shared only between directly connected sites^15,56^, whereas an example of centralised FL aggregation is given in Algorithm 1. Note that aggregation strategies do not necessarily require information about the full model update; clients might chose to share only a subset of the model parameters for the sake of reducing communication overhead, ensure better privacy preservation^10^ or to produce multi-task learning algorithms having only part of their parameters learned in a federated manner.

A unifying framework enabling various training schemes may disentangle compute resources (data and servers) from the *compute plan*, as depicted in Fig. 2. The latter defines the trajectory of a model across several partners, to be trained and evaluated on specific data sets.

#### Algorithm 1

Example of a FL algorithm^16^ via Hub & Spoke (Centralised topology) with FedAvg aggregation^9^.

**Table:** 

**Require:** num_federated_rounds *T*
1: **procedure** AGGREGATING
2: Initialise global model: *W*^(0)^
3: **for** *t* ← 1 ⋯ *T* **do**
4: **for** *client* *k* ← 1 ⋯ *K* **do ** ⊳ *Run in parallel*
5: Send *W*^(*t*−1)^ to client *k*
6: Receive model updates and number of local training iterations $(\Delta {W}_{k}^{(t-1)},{N}_{k})$ from client’s local training with ${{\mathcal{L}}}_{k}({X}_{k};{W}^{(t-1)})$
7: **end for**
8: $${W}^{(t)}\leftarrow {W}^{(t-1)}+\frac{1}{{\sum }_{k}{N}_{k}}{\sum }_{k}({N}_{k}\cdot {W}_{k}^{(t-1)})$$
9: **end for**
10: **return** *W*^(*t*)^
11: **end procedure**

### Challenges and considerations

Despite the advantages of FL, it does not solve all issues that are inherent to learning on medical data. A successful model training still depends on factors like data quality, bias and standardisation^2^. These issues have to be solved for both federated and non-federated learning efforts via appropriate measures, such as careful study design, common protocols for data acquisition, structured reporting and sophisticated methodologies for discovering bias and hidden stratification. In the following, we touch upon the key aspects of FL that are of particular relevance when applied to digital health and need to be taken into account when establishing FL. For technical details and in-depth discussion, we refer the reader to recent surveys^11,12,20^.

#### Data heterogeneity

Medical data is particularly diverse—not only because of the variety of modalities, dimensionality and characteristics in general, but even within a specific protocol due to factors such as acquisition differences, brand of the medical device or local demographics. FL may help address certain sources of bias through potentially increased diversity of data sources, but inhomogeneous data distribution poses a challenge for FL algorithms and strategies, as many are assuming independently and identically distributed (IID) data across the participants. In general, strategies such as *FedAvg*^9^ are prone to fail under these conditions^9,57,58^, in part defeating the very purpose of collaborative learning strategies. Recent results, however, indicate that FL training is still feasible^59^, even if medical data is not uniformly distributed across the institutions^16,17^ or includes a local bias^51^. Research addressing this problem includes, for example, *FedProx*^57^, part-data-sharing strategy^58^ and FL with domain-adaptation^18^. Another challenge is that data heterogeneity may lead to a situation in which the global optimal solution may not be optimal for an individual local participant. The definition of model training optimality should, therefore, be agreed by all participants before training.

#### Privacy and security

Healthcare data is highly sensitive and must be protected accordingly, following appropriate confidentiality procedures. Therefore, some of the key considerations are the trade-offs, strategies and remaining risks regarding the privacy-preserving potential of FL.

Privacy vs. performance: It is important to note that FL does not solve all potential privacy issues and—similar to ML algorithms in general—will always carry some risks. Privacy-preserving techniques for FL offer levels of protection that exceed today’s current commercially available ML models^12^. However, there is a trade-off in terms of performance and these techniques may affect, for example, the accuracy of the final model^10^. Furthermore, future techniques and/or ancillary data could be used to compromise a model previously considered to be low-risk.

Level of trust: Broadly speaking, participating parties can enter two types of FL collaboration:

*Trusted*—for FL consortia in which all parties are considered trustworthy and are bound by an enforceable collaboration agreement, we can eliminate many of the more nefarious motivations, such as deliberate attempts to extract sensitive information or to intentionally corrupt the model. This reduces the need for sophisticated counter-measures, falling back to the principles of standard collaborative research.

*Non-trusted*—in FL systems that operate on larger scales, it might be impractical to establish an enforceable collaborative agreement. Some clients may deliberately try to degrade performance, bring the system down or extract information from other parties. Hence, security strategies will be required to mitigate these risks such as, advanced encryption of model submissions, secure authentication of all parties, traceability of actions, differential privacy, verification systems, execution integrity, model confidentiality and protections against adversarial attacks.

Information leakage: By definition, FL systems avoid sharing healthcare data among participating institutions. However, the shared information may still indirectly expose private data used for local training, e.g., by model inversion^60^ of the model updates, the gradients themselves^61^ or adversarial attacks^62,63^. FL is different from traditional training insofar as the training process is exposed to multiple parties, thereby increasing the risk of leakage via reverse-engineering if adversaries can observe model changes over time, observe specific model updates (i.e., a single institution’s update), or manipulate the model (e.g., induce additional memorisation by others through gradient-ascent-style attacks). Developing counter-measures, such as limiting the granularity of the updates and adding noise^16,18^ and ensuring adequate differential privacy^44^, may be needed and is still an active area of research^12^.

#### Traceability and accountability

As per all safety-critical applications, the reproducibility of a system is important for FL in healthcare. In contrast to centralised training, FL requires multi-party computations in environments that exhibit considerable variety in terms of hardware, software and networks. Traceability of all system assets including data access history, training configurations, and hyperparameter tuning throughout the training processes is thus mandatory. In particular in non-trusted federations, traceability and accountability processes require execution integrity. After the training process reaches the mutually agreed model optimality criteria, it may also be helpful to measure the amount of contribution from each participant, such as computational resources consumed, quality of the data used for local training, etc. These measurements could then be used to determine relevant compensation, and establish a revenue model among the participants^64^. One implication of FL is that researchers are not able to investigate data upon which models are being trained to make sense of unexpected results. Moreover, taking statistical measurements of their training data as part of the model development workflow will need to be approved by the collaborating parties as not violating privacy. Although each site will have access to its own raw data, federations may decide to provide some sort of secure intra-node viewing facility to cater for this need or may provide some other way to increase explainability and interpretability of the global model.

#### System architecture

Unlike running large-scale FL amongst consumer devices such as McMahan et al.^9^, healthcare institutional participants are equipped with relatively powerful computational resources and reliable, higher-throughput networks enabling training of larger models with many more local training steps, and sharing more model information between nodes. These unique characteristics of FL in healthcare also bring challenges such as ensuring data integrity when communicating by use of redundant nodes, designing secure encryption methods to prevent data leakage, or designing appropriate node schedulers to make best-use of the distributed computational devices and reduce idle time.

The administration of such a federation can be realised in different ways. In situations requiring the most stringent data privacy between parties, training may operate via some sort of “honest broker” system, in which a trusted third party acts as the intermediary and facilitates access to data. This setup requires an independent entity controlling the overall system, which may not always be desirable, since it could involve additional cost and procedural viscosity. However, it has the advantage that the precise internal mechanisms can be abstracted away from the clients, making the system more agile and simpler to update. In a peer-to-peer system each site interacts directly with some or all of the other participants. In other words, there is no gatekeeper function, all protocols must be agreed up-front, which requires significant agreement efforts, and changes must be made in a synchronised fashion by all parties to avoid problems. Additionally, in a trustless-based architecture the platform operator may be cryptographically locked into being honest by means of a secure protocol, but this may introduce significant computational overheads.

## Conclusion

ML, and particularly DL, has led to a wide range of innovations in the area of digital healthcare. As all ML methods benefit greatly from the ability to access data that approximates the true global distribution, FL is a promising approach to obtain powerful, accurate, safe, robust and unbiased models. By enabling multiple parties to train collaboratively without the need to exchange or centralise data sets, FL neatly addresses issues related to egress of sensitive medical data. As a consequence, it may open novel research and business avenues and has the potential to improve patient care globally. However, already today, FL has an impact on nearly all stakeholders and the entire treatment cycle, ranging from improved medical image analysis providing clinicians with better diagnostic tools, over true precision medicine by helping to find similar patients, to collaborative and accelerated drug discovery decreasing cost and time-to-market for pharma companies. Not all technical questions have been answered yet and FL will certainly be an active research area throughout the next decade ^12^. Despite this, we truly believe that its potential impact on precision medicine and ultimately improving medical care is very promising.

### Reporting summary

Further information on research design is available in the Nature Research Reporting Summary linked to this article.

## Supplementary information

Reporting Summary Checklist FLAT
